# Towards an Automated Approach for Monitoring Tree Phenology Using Vehicle Dashcams in Urban Environments

**DOI:** 10.3390/s22197672

**Published:** 2022-10-10

**Authors:** Doreen S. Boyd, Sally Crudge, Giles Foody

**Affiliations:** 1 School of Geography, University of Nottingham, Nottingham NG7 2RD, UK; 2 Nottingham Geospatial Institute, University of Nottingham, Nottingham NG7 2RD, UK

**Keywords:** urban tree phenology, dashcam imagery, deep learning, vehicle sensors

## Abstract

Trees in urban environments hold significant value in providing ecosystem services, which will become increasingly important as urban populations grow. Tree phenology is highly sensitive to climatic variation, and resultant phenological shifts have significant impact on ecosystem function. Data on urban tree phenology is important to collect. Typical remote methods to monitor tree phenological transitions, such as satellite remote sensing and fixed digital camera networks, are limited by financial costs and coarse resolutions, both spatially and temporally and thus there exists a data gap in urban settings. Here, we report on a pilot study to evaluate the potential to estimate phenological metrics from imagery acquired with a conventional dashcam fitted to a car. Dashcam images were acquired daily in spring 2020, March to May, for a 2000 m stretch of road in Melksham, UK. This pilot study indicates that time series imagery of urban trees, from which meaningful phenological data can be extracted, is obtainable from a car-mounted dashcam. The method based on the YOLOv3 deep learning algorithm demonstrated suitability for automating stages of processing towards deriving a greenness metric from which the date of tree green-up was calculated. These dates of green-up are similar to those obtained by visual analyses, with a maximum of a 4-day difference; and differences in green-up between trees (species-dependent) were evident. Further work is required to fully automate such an approach for other remote sensing capture methods, and to scale-up through authoritative and citizen science agencies.

## 1. Introduction

Street trees and urban forests influence quality of life for urban citizens. Trees lower air temperature, improve air quality, provide carbon sequestration, intercept precipitation, and regulate run-off and water quality [1]. Urban trees are a necessity for sustaining urban ecology [2]. They provide health benefits to humans, but can also negatively impact human well-being [3]. With an urbanisation projection of 68% for the global population by 2050 [4], there will be an increased dependence on urban trees [5]. Urban trees thus need measurement and monitoring, with their phenological characteristics being a key dataset that is required [6,7].

Phenology has long been monitored through field observations, with this perspective supplemented recently with fixed digital cameras capturing hyper-temporal imagery (e.g., [8,9]). This is useful for object-based (individual tree) analysis, but deployment of fixed sensors across space at fine spatial resolutions is challenging. Sensors on satellites (Earth observation, EO) have also been usefully deployed [10,11], but may not be suitable for individual tree phenological study within the urban context [12]. Sensors on unoccupied aerial vehicles (UAVs) have also been shown to be useful, especially in fusion with satellite data [13,14], but their deployment can be subject to regulatory restrictions. In this paper, we suggest that image data captured by dashcams deployed in vehicles is a new opportunity for urban-situated phenological measurement of trees. Such a remote sensing system offers a ground (road) level view of urban trees and if deployed as part of a crowd-sourced sensor-network or as part of an authoritative network (e.g., work vans, etc.), there is the opportunity to measure the phenology of urban trees beyond the local scale. Furthermore, using dashcams in this way may present an opportunity to collect training and validation data for UAV and satellite-based phenological studies. We present a pilot study to explore the potential of using dashcams for phenological studies. As an initial step this paper focuses on the extraction of tree green-up dynamics in an urban setting using a singular vehicle dashcam sensor.

## 2. Materials and Methods

### 2.1. Data Capture

A survey transect along a road (2000 m in total) in Melksham, UK (51.3704, −2.1376) was driven daily (between 07:45–08:30 GMT) across a 90-day capture period (3 March 2020 to 31 May 2020). Silver Birch, Lime, Chestnut, Hazel, Rowan, and London Plane trees featured, with six locations (Area of Interest—AOIs), comprising single or multiple trees, along the route selected for further study (Table 1). A commercially available dashcam, the Nextbase 322GW (NBDVR322GW), was mounted to a car interior, connected by a 12-24Vdc car power cable, and used for data capture. The sensor resolution was 2.12M Pixels (1936 × 1097) with a recording resolution of 1920 × 1080 (High Definition) at 60 RGB frames per second (fps), with a 140° lens angle and f1.6 focal ratio. Pixel dimensions of a single frame captured were 1366 × 768, with a resolution of 96 dpi and a 24-bit depth.

### 2.2. Automatic Detection of Roadside Trees at Different Phenological Stages

Each of the 90-day video stream frames containing a tree(s) at the six AOIs were manually extracted using the open-source ViedoLAN Client media player—video clips were panned through, and then viewed frame by frame, to derive the frame which provided what was deemed visually to be the optimum view (herein referred to as an image). There was a slight variation to this ‘optimum view’ across the 90-day capture period for each AOI, in part due to some minor movement of the dashcam position, and in part to vehicles obscuring the tree. The six AOIs provided a 523-image dataset (see Table 1). Frames for some days were lost due to connection failure in the car (speed bumps, etc.).

The You Only Look Once (YOLO) v.3 deep learning algorithm [15] was used to automatically extract the tree(s) at each AOI from each of the 523 images. Microsoft’s Visual Object Tagging Tool (VOTT) was used to annotate a subset of images—126 periodically selected across the 90 days (Table 2). For each of the 126 training images, a bounding box was manually drawn around each tree in every image and tagged with the ‘Tree’ class. The YOLOv3 algorithm was deployed on the remaining images to output the original images with bounding boxes and confidence scores for each tree in the image. The performance of the object detection model was evaluated with the Mean Average Precision (mAP) metric on the AOIs used in image analysis [16], which equated to 32 bounding boxes (due to some AOIs featuring multiple trees).

### 2.3. Image Post-Processing and Calculation of Phenological Metrics

Following a visual review of the object detector outputs, the detection results output from the YOLO algorithm was used to filter results to remove any outliers. A total of 64 bounding boxes had a Y minimum coordinate of 0, suggesting that the bounding box had reached the edges of the image. Based on an optimum view test image selection, it was assumed that any coordinates with a Y minimum coordinate had not produced a good enough bounding box for enclosing any trees. A further 62 bounding boxes were removed where the tree class confidence score was <0.30 (determined via a visual analysis, where accurate tree bounding boxes were observed despite a low confidence score of between 0.30 and 0.50). The remaining bounding box coordinates were then used to crop all test images to coordinates, reducing the image size for analysis to a new area of interest—a single tree.

Images were then converted from RGB values to the Hue, Saturation, Value (HSV) colour space to mitigate potential error due to solar variance and brightness in the image, affecting scene illuminance [17,18]. Any images still containing background features (i.e., not the tree), had a user-defined region of interest (ROI) generated. The Green Chromatic Coordinate (GCC) (GCC = green/(red + green + blue)) was then derived for each of all the ~90 image sets of cropped HSV ROI for the detected tree(s).

To further smooth the effects of variable scene illuminance in the data, the temporal data for each tree was calculated as a three-day rolling mean [19]. The start of the leaf-on season was calculated for each tree as per previous studies [9,10]. The image archive was also visually inspected within a validation process in order to ascertain the quality of the automatically computed phenological metric of green-up (following [9]).

## 3. Results

### Tree Detection Evaluation Metrics

**Figure 1:**
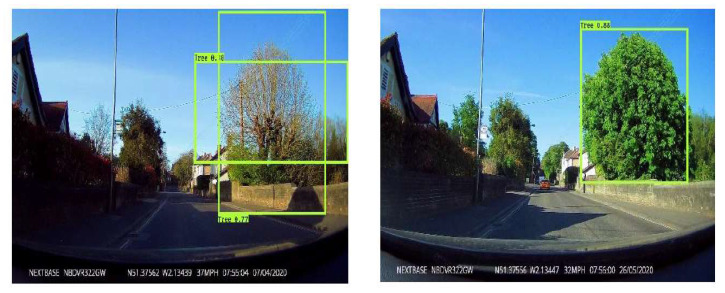
Example comparison of detector outputs between leaf-off and leaf-on trees.

The evaluation dataset showed the YOLOv3 object detector model obtained a mAP accuracy of 87.32% with the IoU threshold set at 50%; and a mAP accuracy of 70.73% with the IoU threshold set at 75%. The detector consistently identified trees in the images; however, there was some variation in the accuracy of the bounding boxes produced. For example, sometimes the detector output multiple bounding boxes for a single tree (23% of test images in AOI_2; 18% in AOI_3; 22% in AOI_4, and 12% in AOI_5). These were straightforward to eliminate from further analysis, as typically where a tree had two or more bounding boxes, one box had a much higher confidence score hence the others were removed. A trend observed across all AOIs was that the detector performed poorer on trees with leaf off, and better with leaf on (see Figure 1 for an example). AOI_1 and AOI_6 were omitted from further analysis following the detector inference, due to the added complexity of obtaining and organising the bounding boxes for image processing where a single image contained >2 trees, particularly due to overlapping bounding boxes.

**Figure 2:**
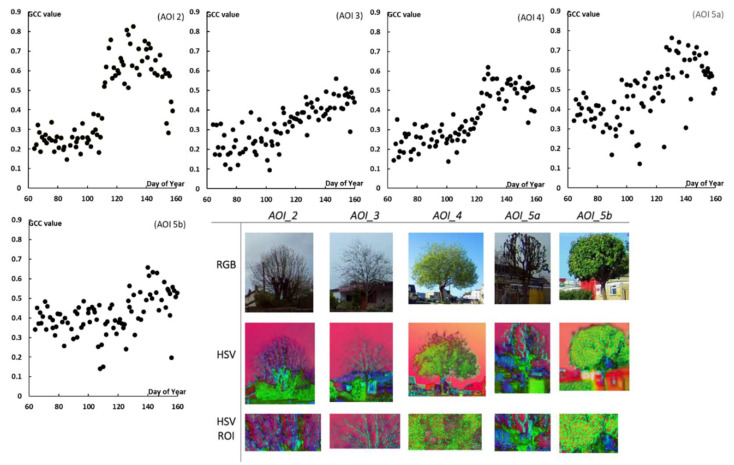
Temporal GCC plotted for AOIs 2, 3, 4, and 5 (two trees) calculated using the ROI HSV; examples of clipped RGB images, the converted HSV image, and the ROI to the HSV colour space, which was then also clipped to new coordinates from each images’ centroid.

The GCC data for HSV ROI images and an example of images used, are plotted for AOI_2, AOI_3, AOI_4, and AOI_5a and b (Figure 2). The phenological signal is clearly well-defined for each AOI—for some AOIs the classic trapezoid phenology profile is displayed; for all AOIs the greenness values are seen to increase rapidly from the start of the time period (March) through to the end of the time period (May). This corresponded to a period of leafing-on in the deciduous trees and associated increased photosynthetic activity. The HSV processing had a stretching effect on the GCC values up until the start of green-up, after which it relatively smoothed the values.

The computed start of green-up (i.e., leaf on) based on the 3-day rolling mean GCC of each HSV ROI is shown in Table 3. Both the Horse Chestnut tree in AOI_2 and the Rowan tree in AOI_3 observed the onset of greenness in early- to mid-April (AOI_2 on the 10 April and AOI_3 on the 14 April. AOI_2 plateaued in greening just four days later on the 18 April, with AOI_3 greening slower, reaching a plateau of greenness on approximately the 9 May. The Plane tree of AOI_4 began green-up later in April, on the 24 April, reaching a maximum greenness on approximately the 29 April. It was difficult to distinguish the onset of greenness in the trees in AOI_5. For both trees in the AOI_5 there was an evident positive relationship between increases in greenness over time, although there were significant fluctuations; however, green-up could be pin-pointed to 23 April for AOI 5 and the 4 May for AOI_5b. In both cases, the fluctuations in the data made it impossible to ascertain if greenness had plateaued, so it was assumed that increased productivity for these trees continued beyond the time period of study. In all cases, these dates of green-up were similar to those obtained by visual analyses with a maximum of a 4-day difference being evident (Table 3).

## 4. Discussion

The potential of dashcams deployed on a vehicle (e.g., a commuter car) to capture high resolution and repeat imagery, subsequently enabling specific object-based analysis on urban trees (extraction of phenological metrics data) has been demonstrated. Of particular note, is that local variability in the green-up start date was captured, which is extremely important considering the heterogeneity of the urban fabric and morphology. The use of dashcams in this way offers a particular advantage over other proximal remote sensing approaches, for example Google Street View data [20,21], in the high temporal frequency afforded (and determined by the user). Further research is required however, and the aspects of this are discussed below.

The mAP accuracies obtained by the YOLOv3 detector were of sufficient quality to calculate the green-up of extracted trees in subsequent image analyses—the YOLOv3 detection algorithm’s capacity to repeatedly detect the same tree from multiple images over time was successful in the main, though further work using the YOLO detector should be undertaken on a much wider range of urban situations and across the whole year. Particular attention should be focused on ensuring the precise intersection of the validation bounding boxes and the detection bounding boxes as this is a determining factor in data consistency and it is important when deriving the greenness metric over time. Inconsistency in the detector precision resulted in error when deriving the greenness metric as this analysis subsequently could not be made to the same portion of the tree. Nonetheless, when used on dashcam data, this deep learning approach did enable object-based analysis, which is not a viable option from other remote sensing data-capture approaches, such as satellite remote sensing techniques, at the temporal resolutions required. Additionally, the deep learning approach largely reduced positioning issues related to the sensor itself. The nature of the dashcam imagery and the urban environment meant that data was subject to changing views; sensor movement and changing road position of the platform (vehicle) due to overtaking cyclists and avoiding roadworks etc., resulted in a specific tree not being repeatedly captured from the same position. However, by obtaining the bounding box coordinates of a given tree, it could be extracted from the data captured by the sensor, and any further analysis could be made solely on the tree, thus the ‘optimum view’ of a tree was not a necessity.

A limitation of using deep learning algorithms is the requirement that the image annotations produce a training dataset for the detector. Pre-existing labelled image catalogues are frequently used for either training or testing (e.g., [22,23]), but this could not be achieved for this application. Unlike previous work, this study aimed to detect trees across a section of the phenological cycle, capturing trees during late winter through to early summer as part of the methodology. Hence, there was a requirement that the training dataset used to train the YOLOv3 detector needed to include annotated images across the phenological timeframe of interest, both trees with leaf off and leaf on, and the growth stages between theses. The performance of the detector on trees with leaf off was comparably weaker than when trees had greened up. It is possible that this is due to the added complexity of visible bare branches, which requires more learning for the detector to work effectively. More research on this is required.

The greenness results reported in this study were largely successful in informing the dates of green-up in different species, and thus better representation of phenological variation could be obtained and incorporated into an improved detector. The difference between the visual analyses and the automated calculation of green-up date (four days maximum) concur with the timings of previous studies focusing on measuring phenological shifts over time (e.g., [24,25]). Developing an optimum model will require balance in the training dataset size in addition to proportional representation of tree phenology. Additionally, when considering the potential reproducibility of the detector in other geographical locations, increased tree species heterogeneity is an advised area of improvement for the training data. Once the desired portion of a tree was extracted from the dashcam RGB data, there was success in capturing the green-up of the tree when applying the Green Chromatic Coordinate metric. Unlike visual observations of phenological events throughout seasons, the collection of repeat digital imagery allows for a quantitative analysis in capturing a change in greenness from the leaf-off to the leaf-on change in trees. The data collected for this study was largely captured during a morning commute, and subsequently there was significant change in the scene illuminance as a result of changing daylight, particularly up until the transition from daylight saving hours to British Summer Time. The HSV colour space used was effective, though further work would be useful.

The potential demonstrated here for low-cost deployment, passive integration of the sensor into a driver’s daily routine, the use of vehicles of both authoritative (e.g., buses and local council vehicles) and non-authoritative bodies (e.g., a commuter), suggests that further research on establishing a dashcam network should be conducted. This should consider how citizen science could be used effectively (e.g., as per Citizens4EO—[26]). Crimmins and Crimmins [27,28] have highlighted the opportunities for engaging the public by encouraging the sharing of repeat photography for capturing phenological events. OpenStreetCam (openstreetcam.org) and Treezilla (treezilla.org) are examples of platforms for collecting and providing free and open data on trees, with the former affording the sharing of dashcam footage and once shared, the automated extraction of phenological metrics could be used to support satellite-based analyses (as training/validation data). A challenge would be to co-ordinate citizen scientists on a larger scale. However, Crooks et al. [29] have recognised that sensor-derived content from crowd-sourced data has great potential in bettering the understanding of city systems, with Park et al. [30] and Rashid et al. [31] acknowledging that sharing dashcam footage will greatly extend urban surveillance. In 2019, the AA Limited, a British motoring association, found that that 24% of respondents already owned a dashcam, with an additional 18% stating that they were seriously considering purchasing one (www.theaa.com/about-us/public-affairs/aa-populus-driver-poll-summaries-2019#june2019; accesses on 10 May 2021). This potential volume of temporal data would be unmatched and offers real scope for the data capture of trees at a national level. Data that is captured by those who make repeated commuter journeys would be of particular interest.

## 5. Conclusions

The dashcam-capture approach presented in this paper points towards an automated process for deriving green-up metrics for urban trees and indicates the scope to ascertain more metrics relating to other aspects of vegetation such as species, health, and size. Further methodological development is required to offer a fully automated process, including improving detector performance, scaling-up capture, and the calculation of the phenological metric of green-up (and potentially senescence). Nonetheless, results obtained in this pilot study suggests that this data capture method, and mobile computing advances in general, could be employed to produce an input dataset, which is required by urban planners for decision making, in addition to acting as a suitable measure for key actions as part of the UN SDG targets (i.e., around sustainable cities (SDG 11); life on land (SDG 15) and climate action (SDG13).

## Figures and Tables

**Table 1:** Description of Area of Interest.

AOI	Contextual Descriptor	Number of Images	Single or Multiple Trees	Species
AOI_1	LimeRow	88	Multiple	Lime
AOI_2	Chestnut Tree	88	Single	Horse Chestnut
AOI_3	Library Tree	87	Single	Rowan
AOI_4	Roundabout Tree	87	Single	Plane
AOI_5	Firestation	87	Multiple	Acer
AOI_6	Semington Rd.	86	Multiple	Birch, Long Leaved Lime

**Table 2:** Days on which a sample of images were annotated for each AOI with the aim of representing the vegetation at various growth stages.

Day of Capture Period	Day of Year	Date (DD/MM/YYYY)
DAY 1	63	3 March 2020
DAY 15	77	17 March 2020
DAY 30	92	1 April 2020
DAY 5	107	16 May 2020
DAY 60	122	1 May 2020
DAY 75	137	16 May 2020
DAY 90	152	31 May 2020

**Table 3:** Start-of-green-up date for each AOI.

AOI	Species of Tree	Dashcam Estimated (DD/MM/YYYY)	Visual Inspection Estimated (DD/MM/YYYY)
2	Horse Chestnut	10 April 2020	13 April 2020
3	Rowan	14 April 2020	10 April 2020
4	Plane	24 April 2020	22 April 2020
5a	Acer	23 April 2020	20 April 2020
5b	Acer	4 May 2020	4 May 2020

## Data Availability

Dashcam data are available on request.
